# Complete revascularization and heart failure risk in acute coronary syndrome across the ejection fraction spectrum: focus on LVEF-dependent effects

**DOI:** 10.3389/fcvm.2026.1830626

**Published:** 2026-05-18

**Authors:** Xiaozhi Luo, Jiaan Nong, Shaowei Xu, Zhongyuan Meng, Xingshou Pan

**Affiliations:** 1 Department of Cardiology, Affiliated Hospital of Youjiang Medical University for Nationalities, Baise, Guangxi, China; 2 Department of Cardiology, Laboratory of the Atherosclerosis and Ischemic Cardiovascular Diseases, Baise, Guangxi, China; 3 Department of Psychosomatic Medicine, Guangxi Zhuang Autonomous Region Brain Hospital, Liuzhou, Guangxi, China

**Keywords:** acute coronary syndrome, complete revascularization, heart failure, left ventricular ejection fraction, multivessel disease

## Abstract

**Objective:**

To investigate the impact of complete revascularization (CR) versus incomplete revascularization (ICR) on the composite outcome of first hospitalization for heart failure (HF) or cardiovascular death in patients with acute coronary syndrome (ACS) and a left ventricular ejection fraction (LVEF) ≥ 40%.

**Methods:**

This retrospective study enrolled 1,834 patients with ACS, multivessel disease, and an LVEF ≥ 40% (767 CR and 1,067 ICR). CR was defined as percutaneous coronary intervention (PCI) of all suitable non-culprit lesions during index hospitalization or within 45 days postdischarge. The primary endpoint was the first occurrence of hospitalization for HF or cardiovascular death. At discharge, almost all patients received dual antiplatelet therapy and guideline-directed medical therapy.

**Result:**

During the follow-up period, 35 of the 767 patients (4.6%) in the CR group and 83 of the 1,067 (7.8%) patients in the ICR group reached the primary endpoint (*p* < 0.001). After adjusting for covariates, CR was associated with a lower risk of the primary endpoint in the population with an LVEF <50% [hazard ratio (HR), 0.46; 95% confidence interval (CI), 0.22–0.96]. This benefit was confined to patients with non-ST-segment elevation ACS (NSTE–ACS) (HR 0.41; 95% CI 0.20–0.83) but was not observed in the ST-segment elevation myocardial infarction (STEMI) subgroup.

**Conclusion:**

CR is associated with a significantly reduced risk of HF hospitalization or cardiovascular death in patients with an LVEF of 40%–50%. This benefit attenuated as the LVEF increased, highlighting a potential LVEF-dependent efficacy. Although these results are promising, the observational nature of this study and the potential for residual confounding necessitate cautious interpretation.

## 1 Introduction

Although percutaneous coronary intervention (PCI) has significantly reduced adverse outcomes in patients with acute coronary syndrome (ACS) (1), its impact on subsequent heart failure (HF) development remains incompletely understood. HF represents one of the major drivers of morbidity and mortality following ACS, substantially increasing hospitalization risk and impairing quality of life. In addition, heart failure with mildly reduced ejection fraction (HFmrEF) and heart failure with preserved ejection fraction (HFpEF) share many clinical features, but their underlying pathophysiology and treatment responses differ (2, 3). A study showed that in dialysis patients with ACS and preserved left ventricular function, angiotensin-converting enzyme inhibitors (ACEIs) or angiotensin II receptor blockers (ARBs) reduced all-cause and cardiovascular mortality. In addition, a more pronounced survival benefit is observed in patients with impaired left ventricular ejection fraction (LVEF) (50%–60%) (4).

Current evidence supports using an LVEF threshold of 50% to define HFmrEF, based on prognostic data showing distinct clinical transitions in the 40%–50% range. This cutoff identifies patients with mild but treatable left ventricular systolic dysfunction, as they demonstrate responsiveness to neurohormonal therapies. However, existing studies have not explored finer LVEF stratifications above 50% (e.g., 50%–60% or >60%), potentially obscuring heterogeneity in treatment responses between patients with HFmrEF and those with HFpEF (5, 6). Extending this concept to complete revascularization (CR), its efficacy in preventing HF across these narrower LVEF subgroups—particularly across the spectrum from mildly reduced (40%–49%) to preserved (≥50%) ejection fraction—remains unclear and may follow a similar pattern of diminishing benefit with higher baseline systolic function. Similarly, while CR reduces mortality in patients with ST-segment elevation myocardial infarction (STEMI) and those with non-ST-segment elevation ACS (NSTE–ACS) who present with an LVEF >40% (1, 7), its efficacy in preventing HF across narrower LVEF subgroups—particularly in patients with mildly reduced or preserved ejection fraction—remains unclear. However, a critical unanswered question is whether the benefit of CR on HF risk is modified by the degree of left ventricular systolic function. Specifically, we hypothesize that the reduction in HF risk associated with CR is strongest among patients with an LVEF <50% and attenuates progressively at higher LVEF levels. Further research is needed to determine whether LVEF modifies the benefits of CR in these populations.

The question whether the efficacy of CR is modified across finer LVEF strata, particularly within the mildly reduced (40%–49%) and preserved (≥50%) ranges, remains unanswered. Therefore, this study aims to evaluate the impact of CR on HF risk in patients with ACS and an LVEF ≥40%, specifically by assessing treatment effects across prespecified LVEF categories (<50%, ≥ 50% to <60%, and ≥60%).

## 2 Methods

### 2.1 Study design and setting

This is a retrospective study that evaluated a strategy of CR (defined as a PCI of all suitable non-culprit lesions) as compared to incomplete revascularization (ICR) in patients with ACS who had undergone successful culprit-lesion PCI (8). Functionally significant non-culprit lesions were defined as those with a visually estimated stenosis >90%. Following PCI, almost all patients received dual antiplatelet therapy and were discharged on guideline-directed medical therapy (1).

### 2.2 Ethical approval and informed consent

The study protocol was approved by the IRB of Youjiang Medical University (2024091301). Written informed consent for participation was not required from the participants or the participants' legal guardians/next of kin in accordance with the national legislation and institutional requirements.

### 2.3 Study cohort and data collection

The CR group underwent a PCI of all functionally significant non-culprit lesions (including those in major proximal coronary arteries and side branches >2.5 mm) either during index hospitalization or within 45 days postdischarge. The ICR group received culprit-only revascularization. Eligible patients met all the following criteria: (1) age ≥ 40 years; (2) a diagnosis of ACS (STEMI or NSTE–ACS); (3) an LVEF ≥ 40%; and (4) ≥  1 non-culprit lesion (stenosis > 90%, diameter ≥ 2.5 mm) (9, 10). The exclusion criteria included the following: (1) a prior diagnosis of HF; (2) a history of HF hospitalization; (3) current use of loop diuretics for the treatment of HF; and (4) the presence of clinical HF signs or symptoms before the occurrence of the index ACS event; or (5) an LVEF < 40% during index hospitalization.

Data on demographic characteristics, clinical data, and key angiographic data were retrospectively collected.

### 2.4 Data documentation

Cardiovascular risk factors were systematically documented, along with prior medical history, including previous MI, revascularization procedures, and stroke events, all of which were verified through a medical record review (1). ACS events were further classified as STEMI, NSTE–ACS, angina pectoris, or unknown classification (11). Hospitalization for HF was defined according to the American College of Cardiology and American Heart Association task force definition: a hospital admission (lasting at least 24 h or extending over a calendar date) with a primary diagnosis of HF, new or worsening symptoms of HF on presentation, objective evidence of new or worsening HF, and initiation or intensification of treatment specifically for HF (12). Major bleeding events were defined as Bleeding Academic Research Consortium type 3 or 5 bleeding events (13).

The LVEF was quantitatively assessed using two-dimensional transthoracic echocardiography and calculated using the biplane Simpson method as follows: [(left ventricular end-diastolic volume left ventricular end-systolic volume)/left ventricular end-diastolic volume] × 100. According to protocol, investigators recorded the LVEF in the electronic case report forms using the most recent echocardiogram obtained within 3 months before screening. For the analysis, the LVEF was categorized as <50%, ≥ 50% to < 60%, and ≥ 60% and analyzed as a continuous variable.

The composite of first hospitalization for HF or cardiovascular death was the primary endpoint. The occurrence of a first hospitalization for HF after the index ACS, confirmed through a review of hospital records, consultation notes, discharge letters, and pertinent laboratory data, along with cardiovascular death and all-cause mortality, constituted the secondary endpoint (1). The adjudication of cardiovascular deaths was based on a review of electronic medical records, clinical visits, and telephonic contact by two cardiovascular experts. Subanalyses of the primary endpoint according to ACS type (STEMI vs. NSTE–ACS) and sensitivity analyses excluding events occurring within the first 30 days were performed.

Results for continuous variables are presented as mean ± standard deviation for normally distributed data and as median and interquartile range for non-normally distributed data, while categorical variables are reported as counts and percentages. Differences in clinical and procedural features between patients with CR and without CR were investigated by performing a *χ*^2^-test for categorical data. For continuous data, normality was checked using the Kolmogorov–Smirnov test. If the distribution was a non-normal one, a Kruskal–Wallis test was used; otherwise, a one-way ANOVA was used. The propensity score (PS) was generated for each patient from a multivariable logistic regression model based on pretreatment covariates as independent variables with complete revascularization as the dependent variable. Pairs of patients were derived using greedy 1:1 matching with a caliper of width of 0.2 SD of the logit of the PS. All variables used for the PS analysis, as well as the *p*-values of their differences in the PS population, are reported in Supplementary Table S1. Moreover, balance was tested using the pstest command as reported in Supplementary Table S1.

For the Cox multivariate analysis, all baseline variables with P < 0.05 between patients with CR and those without CR at univariate analysis were included in the model. The incidence of clinical events was reported as the number of events per 100 patient-years. Logistic regression was used with a treatment-by-LVEF category interaction term to examine whether the treatment effect was modified by the LVEF (14). The effect of CR was calculated with the LVEF included as a continuous variable using a restricted cubic spline. All analyses were performed using IBM SPSS Statistics version 27 and Stata version 18; all statistical tests were two-sided, with P-values with a level of significance (alpha) of < 0.05 to determine statistical significance.

## 3 Results

### 3.1 Population characteristics

Our analysis included 1,834 consecutive patients who presented with ACS and multivessel coronary artery disease. The median follow-up time was 2.22 years (interquartile range, 0.86–3.58 years). Among them, 767 (41.8%) underwent CR, while 1,067 (58.2%) underwent ICR. The baseline characteristics of patients according to the completeness of revascularization are reported in Table 1. Compared with the ICR group, patients with CR were younger, had a higher LVEF and estimated glomerular filtration rate (eGFR), were more likely to undergo staged PCI, and were more frequently prescribed aspirin. During follow-up, 35 (4.6%) patients in the CR group and 83 (7.8%) in the ICR group reached the primary endpoint.

**Table 1:** Baseline characteristics according to baseline treatment.

Characteristics	ICR (*n* = 1,067)	CR (*n* = 767)	*p*-Value
LVEF (IQR)	60 (53–68)	63 (56–70)	<0.001
Age, years (IQR)	64 (55–71)	62 (54–69)	<0.001
Sex, female	243 (22.8%)	166 (21.6%)	0.566
STEMI	440 (41.2%)	280 (36.5%)	0.041
Cardiogenic shock at admission	26 (2.4%)	16 (2.1%)	0.620
eGFR, mL/min/1.73 m^2^ (IQR)	74 (64–88)	79 (67–90)	<0.001
Systolic pressure, mmHg (IQR)	143 (126–160)	142 (125–159)	0.35
History of type 2 diabetes	227 (21.3%)	151 (19.7%)	0.407
History of hypertension	706 (66.2%)	482 (62.8%)	0.141
History of myocardial infarction	40 (3.7%)	23 (3.0%)	0.384
History of PCI	60 (5.6%)	48 (6.3%)	0.569
Statin	1,058 (99.2%)	758 (98.8%)	0.480
Aspirin	1,046 (98.0%)	762 (99.3%)	0.019
Clopidogrel	1,060 (99.3%)	766 (99.9%)	0.092
Beta-blocker intensity	474 (44.4%)	354 (46.2%)	0.463
ACEI	549 (51.5%)	377 (49.2%)	0.331
ARB	85 (8.0%)	55 (7.2%)	0.527
SGLT2 inhibitor	48 (4.5%)	31 (4.0%)	0.635
MRA	187 (17.5%)	96 (12.5%)	0.003
Staged PCI	10 (0.9%)	48 (6.3%)	<0.001
Stenosis (100%)	359 (33.6%)	227 (29.6%)	0.067
Stent characteristics (IQR)	2.0 (1.0–2.0)	2.0 (2.0–2.0)	<0.001
Contrast dose (IQR)	140 (120–170)	141 (120–170)	0.054
Congestion	265 (24.8%)	163 (21.3%)	0.073
Atrial fibrillation	33 (3.1%)	20 (2.6%)	0.541
primary endpoint	83 (7.8%)	35 (4.6%)	<0.001

ACEI, angiotensin-converting enzyme inhibitor; ARB, angiotensin II receptor blocker; CR, complete revascularization; eGFR, estimated glomerular filtration rate; ICR, incomplete revascularization; IQR, interquartile range; LVEF, left ventricular ejection fraction; MRA, mineralocorticoid receptor antagonist; PCI, percutaneous coronary intervention; SD, standard deviation; SGLT2, Sodium-Glucose Cotransporter 2; STEMI, ST-segment elevation myocardial infarction.

### 3.2 Outcomes according to all baseline characteristics

**Figure 1:**
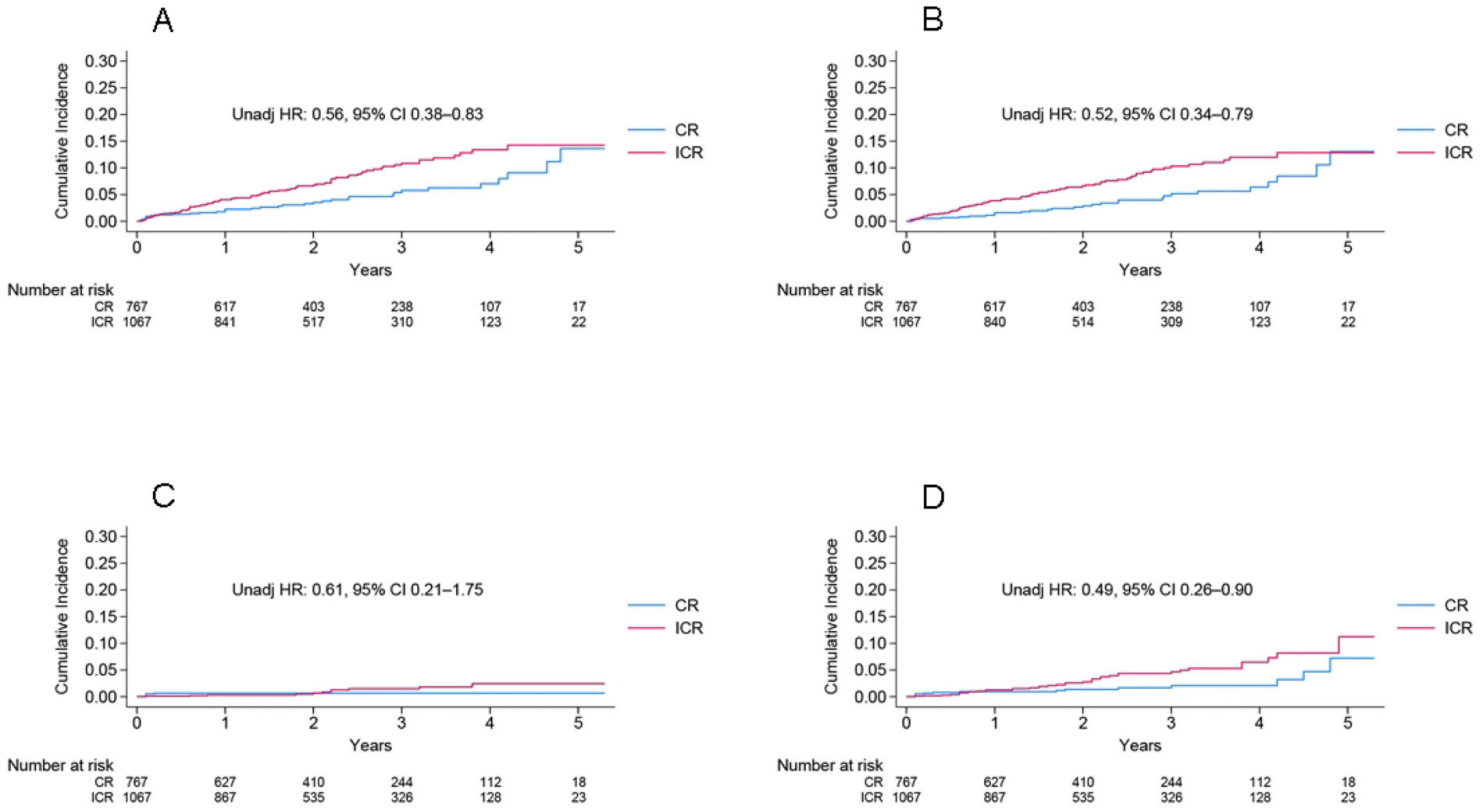
Unadjusted Kaplan−Meier incidence of primary endpoint **(A)**, first HF hospitalization **(B)**, cardiovascular death **(C)**, and all-cause death **(D)**. CR, complete revascularization; HF, heart failure; HR, hazard ratio; ICR, incomplete revascularization.

An *a priori* sample size calculation was not performed because this retrospective analysis included all consecutively eligible patients from the cohort during the study period. However, the total number of primary endpoint events (*n* = 118) was considered sufficient for the analyses conducted. In the unadjusted Kaplan–Meier time-to-event curves, the CR group had a significantly lower cumulative incidence of the primary endpoint compared with the ICR group [hazard ratio (HR), 0.56; 95% confidence interval (CI), 0.38–0.83]; Figure 1 and Table 2. Similar results were observed for the secondary endpoint of the first HF hospitalization (HR 0.52; 95% CI 0.34–0.79), Figure 1] and all-cause death (HR 0.49; 95% CI 0.26–0.90). After a multivariable adjustment, it was found that CR was not statistically significant for the primary endpoint (HR 0.82; 95% CI 0.54–1.25; Table 2).

**Table 2:** Univariate and multivariate Cox regression analyses for primary and secondary outcomes in the overall population and for primary outcomes across ACS subtypes.

Outcomes	No. of events	Unadjusted Cox HR	Adjusted Cox HR
All population			
Primary endpoint	118	0.56 (0.38–0.83)	0.82 (0.54–1.25)
All-cause mortality	52	0.49 (0.26–0.90)	0.70 (0.36–1.37)
STEMI			
Primary endpoint	64	0.98 (0.59–1.62)	1.43 (0.81–2.53)
NSTE-ACS			
Primary endpoint	54	0.30 (0.16–0.59)	0.41 (0.20–0.83)

HR, hazard ratio; NSTE-ACS, non-ST-elevation acute coronary syndrome; STEMI, ST-segment elevation myocardial infarction. Adjusted for the following baseline variables: LVEF, age, eGFR, aspirin, MRA, staged-PCI, and stent characteristics.

### 3.3 Outcomes according to ACS presentation

**Figure 2:**
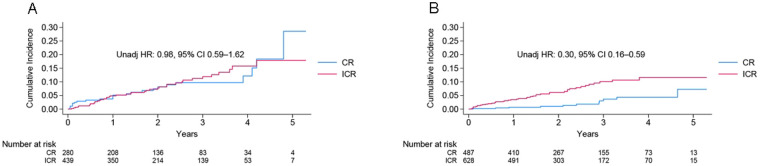
Kaplan−Meier incidence of the primary endpoint in STEMI **(A)** and NSTE-ACS **(B)** according to ACS presentation in the whole population. ACS, acute coronary syndrome; CR, complete revascularization; HR, hazard ratio; ICR, incomplete revascularization; NSTE, non-ST-elevation; STEMI, ST-elevation myocardial infarction.

The incidence of the primary endpoint according to ACS presentation is reported in Figure 2 and Table 2. In the unadjusted Kaplan–Meier time-to-event curves, the CR group had a significantly lower risk of NSTE–ACS compared with the ICR group (HR 0.30; 95% CI 0.16–0.59). After adjustment for covariates, this benefit was confined to patients with NSTE-ACS (HR 0.41; 95% CI 0.20–0.83) and was not observed in the STEMI subgroup. To further compare the risk differences between patient subgroups in NSTE-ACS, we performed a propensity-score matching (PSM) analysis (Supplementary Table S1). The results were also consistent after this analysis (HR 0.38; 95% CI 0.17–0.85)*.* Irrespective of the multivariable adjustment, CR showed no significant difference between the STEMI group and the ICR (HR 0.98; 95% CI 0.59–1.62; adjusted HR 1.43; 95% CI 0.81–2.53).

### 3.4 Outcomes according to LVEF

**Figure 3:**
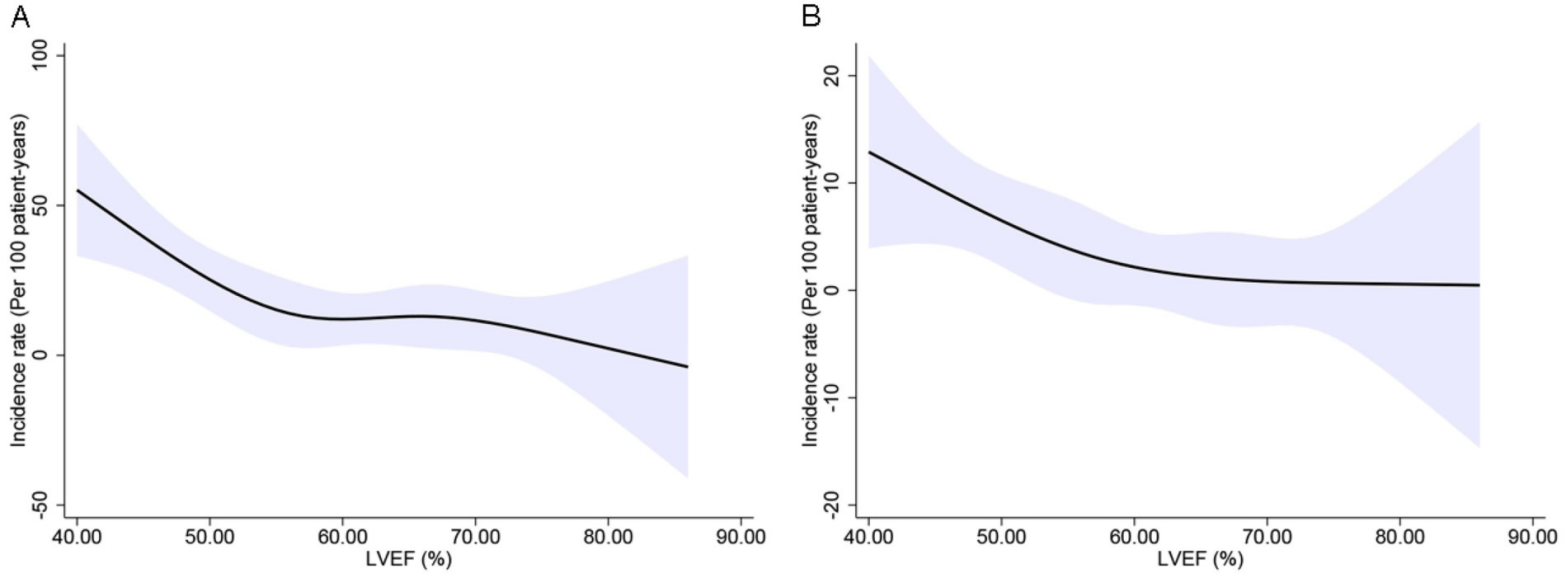
The crude incidence rate of the primary endpoint **(A)** and first HF hospitalization **(B)** per 100 patient-years was calculated using restricted cubic splines with four knots. LVEF, left ventricular ejection fraction; HF, heart failure.

The distribution of the LVEF is displayed in Supplementary Figure S1*.* The baseline characteristics by LVEF group are detailed in Supplementary Table S2. The crude incidence of outcomes by LVEF category is given in Table 3, while baseline LVEF is analyzed as a continuous variable in Figure 3. The rates of the primary and secondary endpoints were highest in patients with a lower LVEF, decreasing across the LVEF range of 50%–60%, and plateauing at ≥60%.

**Table 3:** Risk of outcomes according to the baseline LVEF group.

Outcomes	LVEF group		
	<50% (*n* = 326)	≥50% to <60% (*n* = 462)	≥60% (*n* = 1,046)
First hospitalization for HF or cardiovascular death			
No. of events	50	31	37
Event rate per 100 patient-year (95% CI)	7.55 (5.46–9.64)	3.07 (2.00–4.14)	1.61 (1.10–2.12)
Unadjusted HR (95% CI)	4.69 (3.07–7.18)	1.91 (1.19–3.08)	Ref.
Adjusted HR (95% CI)^a^	3.93 (2.40–6.44)	1.83 (1.12–3.00)	Ref.
First worsening HF events			
No. of events	45	28	34
Event rate per 100 patient-year (95% CI)	6.81 (4.83–8.79)	2.77 (1.75–3.79)	1.48 (0.99–1.97)
Unadjusted HR (95% CI)	4.62 (2.96–7.21)	1.88 (1.14–3.10)	Ref.
Adjusted HR (95% CI)^a^	3.86 (2.30–6.47)	1.81 (1.08–3.04)	Ref.
Cardiovascular death			
No. of events	7	5	4
Event rate per 100 patient-year (95% CI)	1.00 (0.26–1.74)	0.48 (0.07–0.89)	0.17 (0.00–0.34)
Unadjusted HR (95% CI)	5.80 (1.70–19.81)	2.82 (0.76–10.50)	Ref.
Adjusted HR (95% CI)^a^	5.36 (1.34–21.47)	2.81 (0.72–10.93)	Ref.
All-cause mortality			
No. of events	16	11	25
Event rate per 100 patient-year (95% CI)	2.29 (1.17–3.41)	1.06 (0.43–1.69)	1.07 (0.65–1.49)
Unadjusted HR (95% CI)	2.19 (1.17–4.10)	1.00 (0.49–2.04)	Ref.
Adjusted HR (95% CI)^a^	3.05 (1.44–6.44)	1.20 (0.58–2.52)	Ref.

CI, confidence interval; HF, heart failure; HR, hazard ratio; LVEF, left ventricular ejection fraction.

a Adjusted for the following baseline variables: ACEI, ARB, beta-blocker intensity, congestion, contrast dose, MRA, history of hypertension, PCI, SGLT2 inhibitor, STEMI, stenosis, systolic pressure, and woman.

**Figure 4:**
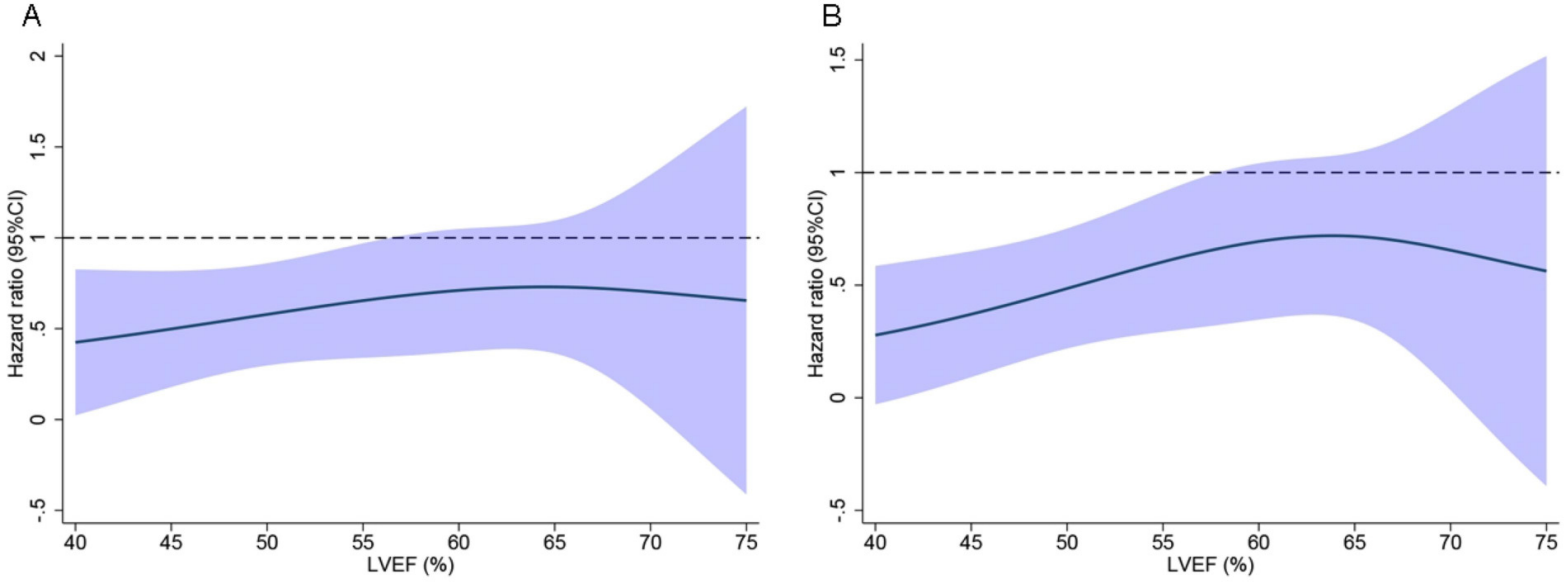
The effect of complete revascularization on the primary endpoint **(A)** and first HF hospitalization **(B)** according to the left ventricular ejection fraction (LVEF), analyzed as a continuous variable, was examined using restricted cubic splines with three knots. CI, confidence interval; HF, heart failure.

The treatment effect of CR compared with ICR on outcomes is detailed by LVEF groups in Table 4, and the LVEF is analyzed as a continuous variable in Figure 4. CR reduced the primary endpoint [LVEF <50% HR, 0.50 (95% CI, 0.26–0.99); ≥ 50% to <60% HR, 0.51 (0.23–1.11); and ≥60% HR, 0.90 (0.47–1.71)].

**Table 4:** Effect of treatment on outcomes according to the baseline LVEF group.

Outcomes	LVEF group						*P* for interaction: categorical	*P* for interaction: continuous
	<50% (*n* = 114)	<50% (*n* = 212)	≥50% to <60% (*n* = 177)	≥50% to <60% (*n* = 285)	≥60% (*n* = 476)	≥60% (*n* = 570)		
	CR	ICR	CR	ICR	CR	ICR		
First hospitalization for HF or cardiovascular death								
No. of events	11	39	8	23	16	21		
Rate (95% CI)	4.62 (2.31–8.27)	9.19 (6.56–12.56)	1.96 (0.85–3.86)	3.81 (2.42–5.72)	1.51 (0.86–2.45)	1.69 (1.05–2.58)		
ARR	8.71%		3.85%		0.38%			
NNT	12		26		263			
HR (95% CI)^a^	0.50 (0.26–0.99)		0.51 (0.23–1.11)		0.90 (0.47–1.71)		0.40	0.22
Adjusted HR (95% CI)^b^	0.46 (0.22–0.96)		0.58 (0.27–1.26)		0.81 (0.40–1.66)		0.39	0.18
First hospitalization for HF								
No. of events	8	37	7	21	15	19		
Rate (95% CI)	3.36 (1.45–6.62)	8.76 (6.20–12.10)	1.71 (0.69–3.53)	3.48 (2.16–5.33)	1.42 (0.79–2.34)	1.53 (0.92–2.38)		
ARR	10.48%		3.71%		0.24%			
NNT	10		27		417			
HR (95% CI) ^a^	0.39 (0.18–0.82)		0.48 (0.21–1.10)		0.93 (0.48–1.82)		0.54	0.26
Adjusted HR (95% CI)^b^	0.34 (0.14–0.80)		0.57 (0.25–1.29)		0.84 (0.40–1.78)		0.18	0.06
Cardiovascular death								
No. of events	3	4	1	4	1	3		
Rate (95% CI)	1.21 (0.25–3.53)	0.89 (0.24–2.27)	0.24 (0.01–1.34)	0.64 (0.17–1.64)	0.09 (0.00–0.53)	0.24 (0.05–0.70)		
ARR	−0.69%		0.88%		0.33%			
NNT	-		114		303			
HR (95% CI)^a^	1.34 (0.29–6.17)		0.39 (0.04–3.54)		0.38 (0.04–3.78)		0.21	0.08
Adjusted HR (95% CI)^b^	1.16 (0.17–7.80)		0.40 (0.08–25.40)		0.41 (0.02–8.30)		0.51	0.23
All-cause mortality								
No. of events	4	12	3	8	7	18		
Rate (95% CI)	1.61 (0.44–4.11)	2.66 (1.38–4.65)	0.73 (0.15–2.13)	1.29 (0.55–2.53)	0.65 (0.26–1.34)	1.42 (0.84–2.25)		
ARR	2.23%		1.21%		1.67%			
NNT	45		83		60			
HR (95% CI)^a^	0.61 (0.19–1.93)		0.53 (0.15–1.90)		0.45 (0.19–1.08)		0.93	0.92
Adjusted HR (95% CI)^b^	0.53 (0.17–1.66)		0.48 (0.03–2.45)		0.37 (0.15–0.96)		0.91	0.86

Event rates are presented per 100 patient-years.

ARR, absolute risk reduction; CR, complete revascularization; ICR, incomplete revascularization; LVEF, left ventricular ejection fraction; NNT, number-needed-to-treat.

a For time to first event outcomes, hazard ratio (HR) and 95% confidence interval (CI) were estimated using Cox regression models.

b Adjusted for the following baseline variables: ACEI, ARB, beta-blocker intensity, congestion, contrast dose, MRA, history of hypertension, PCI, SGLT2 inhibitor, STEMI, stenosis, systolic pressure, and woman.

Similar outcomes were presented in the first hospitalization for HF [LVEF <50% HR, 0.39 (95% CI 0.18–0.82); ≥50% to <60% HR, 0.48 (0.21–1.10); and ≥60% HR 0.93 (0.48–1.82)]. However, the CR group had a non-significantly lower cumulative incidence of cardiovascular death and all-cause mortality across the whole LVEF compared with the ICR group.

After the multivariable adjustment, CR also reduced the risk of the primary endpoint [LVEF <50% adjusted HR 0.46; 95% CI 0.22–0.96; ≥ 50% to <60% adjusted HR 0.58 (0.27–1.26); and ≥60% adjusted HR 0.81 (0.40–1.66)] and the first hospitalization for HF [LVEF <50% adjusted HR 0.34; 95% CI 0.14–0.80; ≥50% to <60% adjusted HR 0.57 (0.25–1.29); and ≥60% adjusted HR 0.84 (0.40–1.78)]. In LVEF ≥60%, the CR group had a lower cumulative incidence of all-cause mortality compared with the ICR group [LVEF <50% adjusted HR 0.53; 95% CI 0.17–1.66; ≥50% to <60% adjusted HR 0.48 (0.03–2.45); and ≥60% adjusted HR 0.37 (0.15–0.96)]. The effect of CR on reducing the risk of the primary endpoint and secondary endpoint was not modified by the LVEF when analyzed in categories (p_interaction_ > 0.05) or as a continuous variable (*p*_interaction_ > 0.05).

### 3.5 Sensitivity analyses

Sensitivity analyses excluding events in the first 30 days are presented in Supplementary Tables S3–S5, which also showed that CR is associated with a significantly reduced risk of HF hospitalization or cardiovascular death in patients with NSTE–ACS (adjusted HR 0.38; 95% CI 0.18–0.80) or LVEF ≥40% [LVEF <50% adjusted HR 0.33; 95% CI 0.14–0.78]; ≥50% to <60% adjusted HR 0.54 (0.24–1.22); and ≥60% adjusted HR 0.66 (0.30–1.43)].

### 3.6 Safety outcomes according to LVEF category

The occurrence of safety outcomes according to LVEF category is detailed in Supplementary Table S6. Patients in the ICR group had more frequent increases in bleeding than those in the CR group, and this did not differ significantly across LVEF groups.

## 4 Discussion

In this study, we sought to evaluate the impact of CR on the incidence of the first hospitalization for HF or cardiovascular death in patients with ACS and LVEF ≥40% undergoing PCI. We found that CR was associated with a significantly reduced risk of HF hospitalization or cardiovascular death in patients with NSTE–ACS. A further analysis by LVEF showed that CR reduced the risk of the primary endpoint in the LVEF <50% population.

Consistent with previous research analysis stratified by LVEF, we found that the CR group had a reduced incidence of the primary endpoint (1). In recent years, there has been increasing evidence supporting CR in patients with STEMI, and CR of non-culprit lesions has been clearly associated with a reduced rate of death, MI, the composite of cardiovascular death or HF hospitalization, and ischemia-driven revascularization in patients with multivessel disease (1, 15, 16). In our study, the subgroup analysis showed that CR reduced the primary endpoint in patients with NSTE–ACS, while no significant benefit was observed in STEMI. The significant benefit observed in the NSTE–ACS subgroup is attributed to a higher applicability of staged management, a disease profile characterized by multifocal instability amenable to systemic treatment, and the intervention's mechanism aligning with plaque stabilization and secondary prevention. The lack of benefit in STEMI may stem from the primacy of acute reperfusion, the higher rate of completed infarction, and the shorter-term focus of the initial intervention.

The crude event rates peaked in patients with the lowest LVEF and plateaued above 60%. This observation aligns with known pathophysiological mechanisms: In ACS with reduced LVEF (<50%), the inverse linear association between LVEF and adverse outcomes underscores the central role of systolic dysfunction and progressive ventricular remodeling. In addition, non-culprit lesions indeed may jeopardize a large amount of myocardium, which can be exposed to chronic ischemia and therefore enter a state of hibernation. This may also entail a negative remodeling of the involved non-culprit segment (17, 18). This is further supported by recent proteomic evidence showing that dysregulation of inflammatory and metabolic pathways (e.g., cathepsin L and serum amyloid A1) in post-MI heart failure contributes to ventricular dysfunction and disease progression (19). In preserved LVEF (≥60%), the stabilized event rates suggest alternative dominant pathways (e.g., microvascular dysfunction or diastolic impairment). The diminished benefit of CR in patients with LVEF ≥60% likely reflects the distinct pathophysiology of heart failure in this group. Here, HF events are less driven by obstructive epicardial disease and more by microvascular dysfunction, diastolic impairment, and comorbidities. A PCI of non-culprit lesions may therefore have less impact on preventing HF hospitalization, as it does not address these primary mechanisms of HFpEF.

The treatment effect of CR on the primary endpoint appeared to be significant in patients with an LVEF between 40% and <50% upon an assessment of the categories analysis; however, a further analysis revealed that the effect was not modified by the LVEF when analyzed in categories or as a continuous variable. These data support CR as a foundational therapy for patients with ACS across the spectrum of LVEF (≥40%).

Although robust evidence from landmark trials has established the benefits of CR in ACS patients with multivessel disease (1), critical knowledge gaps persist regarding the effects on LVEF (20). Few studies have systematically evaluated the efficacy of CR across the full ejection fraction spectrum (including LVEF <40%, 40%–49%, and≥50% subgroups). However, the effects of other therapies in ≥40% of patients with LVEF have been confirmed. For instance, recent studies found that while neurohormonal therapies maintained efficacy across all LVEF strata ≥40% (21), the clinical benefits of spironolactone diminished with increasing LVEF. Our study fills this research gap by evaluating CR outcomes stratified by LVEF categories (<50%, ≥50% to <60%, ≥60%), a prespecified analysis absent in prior CR trials, including CORALYS (1). Consistent with neurohormonal agents such as finerenone, our data suggest that CR provides directionally consistent benefits across LVEF categories.

In our analysis, the benefit of CR was evident in patients with 40% ≤ LVEF < 50%. Data about the impact of CR in patients with ACS and preserved LVEF are scant, especially in the context of LVEF>50%. The CULPRIT shock trial showed that an immediate multivessel PCI in patients with ACS and acute cardiogenic shock was associated with higher rates of death and renal failure as compared to culprit-only PCI, but real-world data reveal contrasting findings (22–24). However, in our analysis, only a small proportion of patients presented with cardiogenic shock. Our findings contrast with the harm associated with multivessel PCI in cardiogenic shock (as seen in CULPRIT–SHOCK), which is possibly attributed to the fact that our cohort comprised predominantly hemodynamically stable patients (only 2.3% presented with shock). Thus, the benefits of CR observed here apply to the broader, stable ACS population with multivessel disease and LVEF ≥40%.

In our study, almost all patients received dual antiplatelet therapy with aspirin and clopidogrel, and ticagrelor was administered only in a small subset. Current evidence on these two purinergic P2Y Receptor 12 receptor inhibitors remains controversial: some studies have demonstrated the superiority of ticagrelor over clopidogrel in terms of efficacy (25, 26). However, a cohort study conducted in China (27) indicated that ticagrelor demonstrated superior efficacy over clopidogrel in patients with low bleeding risk.

This study is subject to the usual limitations of a retrospective study; the data on the proportion of non-culprit lesions treated based on physiology vs. angiography and the residual SYNTAX score or a comparable metric of anatomic completeness were not systematically recorded. For the cardiovascular mortality outcome, the limited number of events may introduce considerable uncertainty into the estimation of the absolute risk reduction. Because of logistical constraints, we were unable to collect long-term follow-up data and had no model such as a built-in time-updated medication model. Moreover, while CABG occurrence was sometimes noted, the exact date of surgery was missing in most cases, precluding any time-to-event or time-updated analysis, and the baseline variable of the utility of CABG was missing. Furthermore, this was a non-randomized study in which group allocation was based on symptom severity, patient preference, and physician judgment; selection bias may therefore exist. Although we adjusted for measured confounders, residual confounding cannot be ruled out, and the findings should be interpreted with caution.

In conclusion, our analysis suggests that in patients with ACS, CR reduced HF risk in patients with NSTE–ACS or in patients with an LVEF of 40%–50%. The benefit attenuated as the LVEF increased, highlighting potential LVEF-dependent efficacy. While these results are promising, the observational nature of our study and potential residual confounding necessitate cautious interpretation. Future research can explore the mechanisms underlying LVEF-specific treatment responses and validate our findings in prospective cohorts.

## Data Availability

The raw data supporting the conclusions of this article will be made available by the authors, without undue reservation.
