# Supplementary material for: Complete revascularization and heart failure risk in acute coronary syndrome across the ejection fraction spectrum: focus on LVEF-dependent effects
Source: Front Cardiovasc Med. 2026 May 18;13:1830626. doi: 10.3389/fcvm.2026.1830626 (PMC13223025; doi:10.3389/fcvm.2026.1830626)
Supplement: Supplementary file 1 [file Datasheet1.docx]

**The criteria of HF hospitalization:**

Dyspnea (dyspnea with exertion, dyspnea at rest, orthopnea, paroxysmal nocturnal dyspnea, nocturnal cough in supine position, tachypnea);Decreased exercise tolerance (reduced ability to perform activities that involve dynamic movement of large skeletal muscles because of symptoms of dyspnea or fatigue); Fatigue (usually described as feeling a lack of energy and motivation in both mental and physical activities, easily tiring and not being able to complete usual activities, and sometimes accompanied by dizziness, lightheadedness); Worsened end-organ perfusion (worsening cerebral, renal, liver, abdominal or gastrointestinal, peripheral circulatory function manifested by symptoms such as dizziness, lightheadedness, syncope, confusion, altered mental status, restlessness, decline in cognitive state, nausea, vomiting, abdominal pain, abdominal fullness, abdominal discomfort or abdominal tenderness, cold clammy extremities, discoloration of extremities or lips, jaundice, pain in extremities, reduced urine output, darkening of urine color, chest pain, and/or palpitations); or other symptoms of volume overload (swelling of lower extremities; swelling or indentation of pressure marks in areas of fluid accumulation such as the legs, ankles, or lower back; an increase in abdominal girth, right-sided abdominal fullness, discomfort, or tenderness; an increase in body weight; oozing and development of skin breakdown in lower extremities).

Natriuretic peptides: In acute decompensated HF, natriuretic peptide levels are usually significantly elevated (e.g., BNP >500 pg/mL or NT-proBNP >2,000 pg/mL).

IV diuretic use: **Required intravenous diuretic therapy during the hospitalization as part of the management directed at heart failure.**

Imaging findings： Radiological evidence of pulmonary congestion (CXR or other imaging modality such as CT or MRI with evidence of pulmonary venous or alveolar congestion, interstitial or pulmonary edema, pleural effusion, or cephalization of venous flow. It is important to note that CXR may also reflect evidence of cardiomegaly.

**The clarification on adjudication and blinding:**

All suspected primary endpoint events (heart failure hospitalization and cardiovascular death) were **centrally and independently adjudicated** by a **blinded Clinical Events Committee (CEC)**.

**Adjudication Process: Event Identification:** Potential endpoint events were identified by site investigators and reported to the coordinating center.

**Document Collection:** The coordinating center collected all relevant source documents (e.g., hospitalization records, discharge summaries, laboratory results, imaging reports, death certificates, autopsy reports).

**Blinded Review:** All personally identifiable information and treatment group assignments were redacted from the collected documents.

**CEC Review:** The blinded CEC, composed of independent clinicians (e.g., cardiologists) not involved in the trial conduct or analysis, reviewed the anonymized case materials according to pre-specified, standardized definitions based on ACC/AHA data standards.

**Final Classification:** The CEC determined whether the event met the protocol-defined criteria for the primary endpoint (HF hospitalization or CV death) and assigned the official classification. Only events confirmed by the CEC were included in the final efficacy analysis.

**Blinding:** The **CEC members** were fully blinded to treatment allocation throughout the adjudication process. **Site investigators and study personnel** involved in patient care and data entry were not involved in the endpoint adjudication decision-making.

# Figure legend


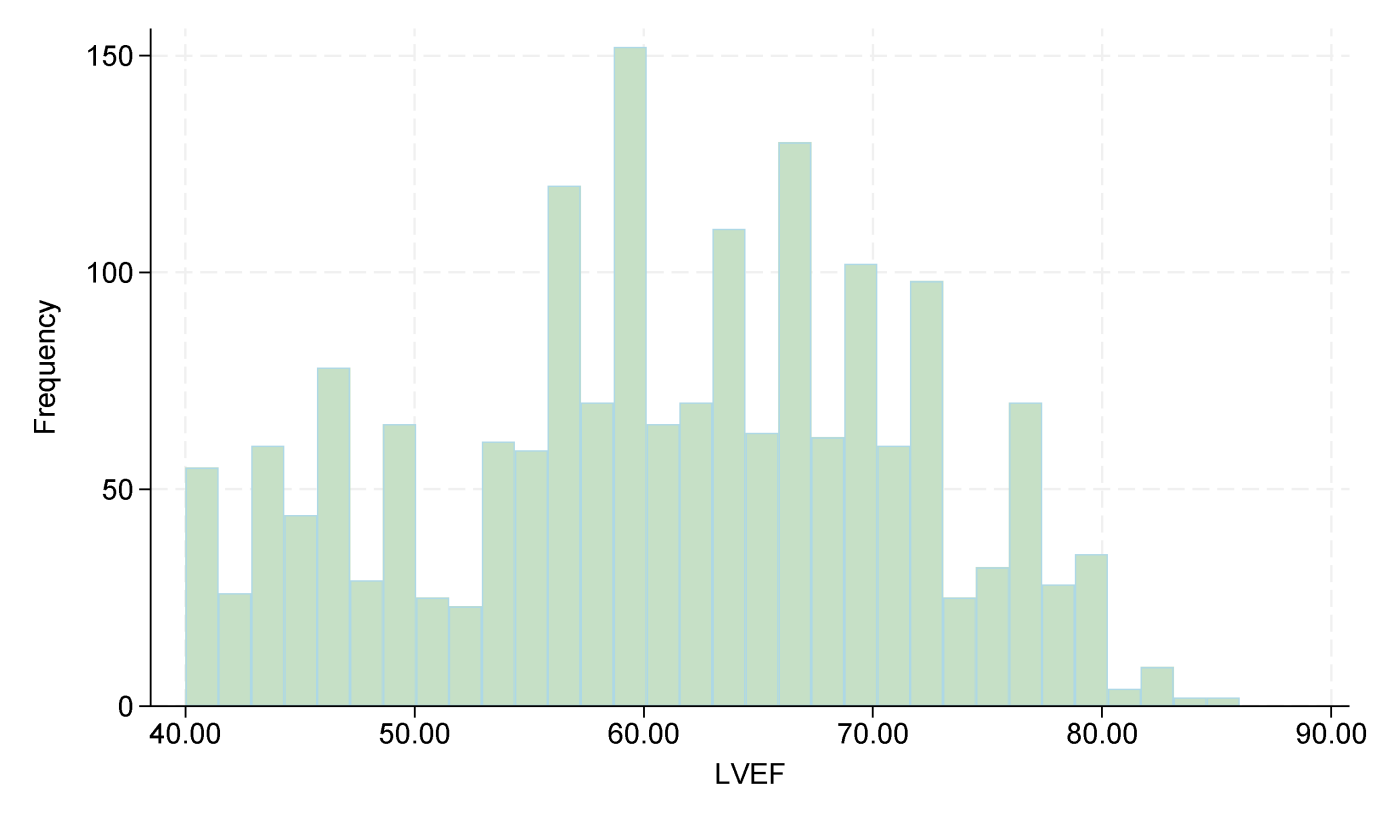


**Fig. S1** Distribution of baseline left ventricular ejection fraction. Abbreviations: LVEF, left ventricular ejection fraction.

# Table legend

**Table S1** Baseline characteristics, PSM and PSM adjusted HR of the primary outcomes in NSTE-ACS

|  | Original cohort (before PSM) | | *p* value | SMD | Matched cohort (after PSM) | | *p* value | SMD | PS adjusted Cox HR |
| --- | --- | --- | --- | --- | --- | --- | --- | --- | --- |
|  | ICR (*n* = 627) | CR (*n* = 487) |  |  | ICR (*n* = 338) | CR (*n* = 338) |  |  |  |
| LVEF | 63 (56-70) | 66 (60-71) | <0.001 | 0.26 | 65 (58-71) | 65 (59-71) | 0.921 | 0.023 | 0.38 (0.17-0.85) |
| Age, years (IQR) | 65 (56-72) | 61 (54-69) | <0.001 | 0.29 | 63 (54-69) | 63 (55-70) | 0.449 | 0.067 |  |
| eGFR, mL/min/1.73 m² (IQR) | 72 (60-84) | 76 (66-85) | <0.001 | 0.24 | 76 (66-89) | 76 (65-84) | 0.347 | 0.093 |  |
| MRA | 75 (12.0) | 32 (6.6) | 0.003 | 0.19 | 24 (7.1) | 27 (8.0) | 0.771 | 0.034 |  |
| Staged-PCI | 4 (0.6) | 40 (8.2) | <0.001 | 0.38 | 3 (0.9) | 7 (2.1) | 0.339 | 0.098 |  |
| Stent characteristics | 2 (1.0-2.0) | 2 (2.0-2.9) | <0.001 | 0.54 | 2 (2.0-3.0) | 2 (2.0-2.0) | 0.339 | 0.024 |  |

*Abbreviations: CR, complete revascularization; eGFR, estimated glomerular filtration rate; ICR, incomplete revascularization; lvef, left ventricular ejection fraction;* *MRA,* ***M****ineralocorticoid****R****eceptor****A****ntagonist; PSM, propensity score matching; SMD, standardized mean differences.*

**Table S2** Baseline characteristics according to baseline left ventricular ejection fraction group

| Characteristics | LVEF group | | | *p value* |
| --- | --- | --- | --- | --- |
|  | <50%  (*n* = 326) | ≥50% to <60%  (*n* = 462) | ≥60%  (*n* = 1046) |  |
| Age, years (IQR) | 63 (53-71) | 62 (54-69) | 63 (55-70) | 0.420 |
| Sex, female | 68 (20.9%) | 85 (18.4%) | 256 (24.5%) | 0.026 |
| STEMI | 216 (66.3%) | 235 (50.9%) | 269 (25.7%) | <0.001 |
| Cardiogenic shock at admission | 12 (3.7%) | 9 (1.9%) | 21 (2.0%) | 0.180 |
| eGFR, mL/min/1.73 m² (IQR) | 78 (64-90) | 76 (64-90) | 76 (65-88) | 0.493 |
| Systolic pressure, mmHg (IQR) | 136 (120-154) | 142 (123-159) | 145 (128-161) | <0.001 |
| History of type 2 diabetes | 64 (19.6%) | 89 (19.3%) | 225 (21.5%) | 0.756 |
| History of hypertension | 183 (56.1%) | 277 (60.0%) | 728 (69.6%) | <0.001 |
| History of myocardial infarction | 12 (3.7%) | 17 (3.7%) | 34 (3.3%) | 0.882 |
| History of PCI | 10 (3.1%) | 23 (5.0%) | 75 (7.2%) | 0.014 |
| ACEi/ARB | 187 (57.4%) | 250 (54.1%) | 590 (56.4%) | 0.225 |
| Statin | 321 (98.5%) | 458 (99.1%) | 1037 (99.1%) | 0.537 |
| Aspirin | 323 (99.1%) | 457 (98.9%) | 1028 (98.3%) | 0.526 |
| Clopidogrel | 324 (99.4%) | 460 (99.6%) | 1042 (99.6%) | 0.858 |
| Beta-blocker intensity | 174 (53.4%) | 225 (48.7%) | 429 (41.0%) | <0.001 |
| ACEI | 186 (57.1%) | 228 (49.4%) | 512 (48.9%) | 0.032 |
| ARB | 17 (5.2%) | 29 (6.3%) | 94 (9.0%) | 0.036 |
| SGLT2 inhibitor | 25 (7.7%) | 21 (4.5%) | 33 (3.2%) | 0.002 |
| MRA | 129 (39.6%) | 86 (18.6%) | 68 (6.5%) | <0.001 |
| Staged-PCI | 6 (1.8%) | 14 (3.0%) | 38 (3.6%) | 0.267 |
| Stenosis (100%) | 134 (41.1%) | 180 (39.0%) | 272 (26.0%) | <0.001 |
| Stent characteristics (IQR) | 2 (1-2) | 2 (1-2) | 2 (2-2) | 0.809 |
| **Contrast dose, mL (**IQR**)** | 130 (100-160) | 140 (120-170) | 140 (120-170) | 0.002 |
| Congestion | 126 (38.7%) | 133 (28.8%) | 169 (16.2%) | <0.001 |
| Atrial fibrillation | 12 (3.7%) | 11 (2.4%) | 30 (2.9%) | 0.561 |

*Abbreviations: ACEI, angiotensin-converting enzyme inhibitor; ARB, angiotensin II receptor blocker; eGFR, estimated glomerular filtration rate; IQR, interquartile range; LVEF, left ventricular ejection fraction; MRA,* ***M****ineralocorticoid****R****eceptor****A****ntagonist; PCI, percutaneous coronary intervention; SD, standard deviation; SGLT2,* ***Sodium-Glucose Cotransporter 2;*** *STEMI, ST-segment elevation myocardial infarction.*

**Table S3** Univariate and multivariate Cox regression analyses for primary and secondary outcomes in the overall population and for primary outcomes across ACS subtypes

|  | NO. of events | Unadjusted Cox HR | Adjusted Cox HR |
| --- | --- | --- | --- |
| All population |  |  |  |
| Primary end point | 108 | 0.46 (0.30-0.71) | 0.65 (0.41-1.02) |
| All-cause mortality | 47 | 0.36 (0.18-0.72) | 2.96 (1.46-6.00) |
| STEMI |  |  |  |
| Primary end point | 58 | 0.74 (0.42-1.29) | 1.04 (0.56-1.92) |
| NSTE-ACS |  |  |  |
| Primary end point | 50 | 0.29 (0.15-0.59) | 0.38 (0.18-0.80) |

*Abbreviations: HF, heart failure; HR, hazard ratio; NSTE-ACS, non‐ST‐elevation acute coronary syndrome; STEMI, ST-segment elevation myocardial infarction. Adjusted for the following baseline variables: LVEF, age, eGFR, aspirin, MRA, staged-PCI, stent characteristics*.

**Table S4** Risk of outcomes according to the baseline LVEF group

| Outcomes | LVEF group | | |
| --- | --- | --- | --- |
|  | <50% (*n* = 322) | ≥50% to <60% (*n* = 460) | ≥60% (*n* = 1042) |
| First hospitalization for HF or cardiovascular death | | | |
| NO. of events | 46 | 29 | 33 |
| Event rate per 100 patient-year (95% CI) | 6.95 (4.94-8.96) | 2.87 (1.83-3.91) | 1.43 (0.94-1.92) |
| Unadjusted HR (95% CI) | 4.87 (3.12-7.62) | 2.01 (1.22-3.31) | Ref. |
| Adjusted HR (95% CI) ^a^ | 4.18 (2.49-7.03) | 1.97 (1.17-3.31) | Ref. |
| First worsening HF events | | | |
| NO. of events | 43 | 28 | 31 |
| Event rate per 100 patient-year (95% CI) | 6.52 (4.57-8.46) | 2.77 (1.74-3.80) | 1.35 (0.87-1.82) |
| Unadjusted HR (95% CI) | 4.86 (3.06-7.72) | 2.06 (1.24-3.44) | Ref. |
| Adjusted HR (95% CI) ^a^ | 4.12 (2.41-7.03) | 2.02 (1.18-3.42) | Ref. |
| Cardiovascular death | | | |
| NO. of events | 5 | 3 | 3 |
| Event rate per 100 patient-year (95% CI) | 0.72 (0.09-1.35) | 0.29 (0.00-0.62) | 0.13 (0.00-0.27) |
| Unadjusted HR (95% CI) | 5.61 (1.34-23.49) | 2.24 (0.45-11.12) | Ref. |
| Adjusted HR (95% CI) ^a^ | 6.36 (1.89-34.09) | 2.66 (0.50-14.08) | Ref. |
| All-cause mortality | | | |
| NO. of events | 14 | 9 | 24 |
| Event rate per 100 patient-year (95% CI) | 2.01 (0.96-3.07) | 0.87 (0.30-1.44) | 1.03 (0.62-1.44) |
| Unadjusted HR (95% CI) | 2.01 (1.04-3.88) | 0.85 (0.40-1.83) | Ref. |
| Adjusted HR (95% CI) ^a^ | 3.11 (1.40-6.90) | 1.09 (0.49-2.43) | Ref. |

*Abbreviations: CI, confidence interval; HF, heart failure; HR, hazard ratio; LVEF, left ventricular ejection fraction.a Adjusted for the following baseline variables: ACEI, ARB, beta-blocker intensity, congestion,* ***contrast dose, MRA,*** *history of hypertension, PCI, SGLT2 inhibitor, STEMI, stenosis , systolic pressure, and woman.*

**Table S5** Effect of treatment on outcomes according to baseline LVEF group

| Outcomes | LVEF group | | | | | | *P* vaule categorical LVEF group *treatment interaction | *P* vaule continuous LVEF group *treatment interaction |
| --- | --- | --- | --- | --- | --- | --- | --- | --- |
|  | <50%  (*n* = 111) | <50%  (*n* = 211) | ≥50% to <60%  (*n* = 176) | ≥50% to <60%  (*n* = 284) | ≥60%  (*n* = 473) | ≥60%  (*n* = 569) |  |  |
|  | CR | ICR | CR | ICR | CR | ICR |  |  |
| First hospitalization for HF or cardiovascular death | | | | | | |  |  |
| NO. of events | 8 | 38 | 7 | 22 | 13 | 20 |  |  |
| Rate (95% CI) | 3.37 (1.03-5.70) | 8.96 (6.11-11.81) | 1.72 (0.45-2.99) | 3.65 (2.13-5.18) | 1.23 (0.56-1.90) | 1.61 (0.90-2.31) |  |  |
| ARR | 5.59% | | 1.93% | | 0.38% | |  |  |
| NNT | 18 | | 52 | | 263 | |  |  |
| HR (95% CI) ^a^ | 0.38 (0.18-0.80) | | 0.46 (0.20-1.05) | | 0.76 (0.38-1.53) | | 0.38 | 0.09 |
| Adjusted HR (95% CI) ^b^ | 0.33 (0.14-0.78) | | 0.54 (0.24-1.22) | | 0.66 (0.30-1.43) | | 0.32 | 0.07 |
| First hospitalization for HF | | | | | | |  |  |
| NO. of events | 7 | 36 | 7 | 21 | 13 | 18 |  |  |
| Rate (95% CI) | 2.95 (0.76-5.13) | 8.52(5.74-11.31) | 1.72 (0.44-2.99) | 3.49 (2.00-4.98) | 1.23 (0.56-1.89) | 1.45 (0.78-2.12) |  |  |
| ARR | 5.57% | | 1.77% | | 0.22% | |  |  |
| NNT | 18 | | 57 | | 455 | |  |  |
| HR (95% CI) ^a^ | 0.35  (0.16-0.77) | | 0.48 (0.21-1.10) | | 0.85 (0.42-1.72) | | 0.25 | 0.06 |
| Adjusted HR (95% CI) ^b^ | 0.30 (0.12-0.76) | | 0.57 (0.25-1.30) | | 0.75 (0.34-1.63) | | 0.21 | 0.05 |
| All-cause mortality | | | | | | |  |  |
| NO. of events | 2 | 12 | 2 | 7 | 6 | 18 |  |  |
| Rate (95% CI) | 0.82 (0.00-1.95) | 2.66 (1.16-4.17) | 0.49 (0.00-1.16) | 1.12 (0.29-1.96) | 0.56 (0.11-1.01) | 1.43 (0.77-2.08) |  |  |
| ARR | 1.84% | | 0.63% | | 0.87% | |  |  |
| NNT | 54 | | 159 | | 115 | |  |  |
| HR (95% CI) ^a^ | 0.31 (0.07-1.41) | | 0.39 (0.09-1.71) | | 0.39 (0.16-0.99) | | 0.95 | 0.48 |
| Adjusted HR (95% CI) ^b^ | 0.27 (0.06-1.29) | | 0.34 (0.06-2.01) | | 0.30 (0.11-0.81) | | 0.87 | 0.55 |

*Event rates are presented per 100 patient-years. Abbreviations: ARR, absolute risk reduction; CR, complete revascularization; ICR, incomplete revascularization; LVEF, left ventricular ejection fraction; NNT, number-needed-to-treat. a For time to first event outcomes, hazard ratio (HR) and 95% confidence interval (CI) were estimated using Cox regression models. b Adjusted for the following baseline variables: ACEI, ARB, beta-blocker intensity, congestion,* ***contrast dose, MRA,*** *history of hypertension, PCI, SGLT2 inhibitor, STEMI, stenosis , systolic pressure, and woman.*

**Table S6**. Safety outcomes according to baseline LVEF group

| Outcomes | LVEF group | | | | | | *p*-interaction |
| --- | --- | --- | --- | --- | --- | --- | --- |
|  | <50% (n=326) | | ≧50% to <60% (n=462) | | ≧60% (n=1046) | |  |
|  | CR  (n=114) | ICR  (n=212) | CR  (n=177) | ICR  (n=285) | CR  (n=476) | ICR  (n=570) |  |
| Stroke | | | | | | | |
| No.(%) | 5 /114(4.4) | 4/212 (1.9) | 5/177 (2.8) | 8/285 (2.8) | 9/476 (1.9) | 24/570 (4.2) |  |
| OR(95%CI) | 2.41 (0.64-9.07) | | 1.01 (0.33-3.08) | | 0.45 (0.21-0.97) | | 0.94 |
| Bleeding | | | | | | | |
| No.(%) | 2 /114(1.8) | 10/212 (4.7) | 2/177 (1.1) | 6/285 (2.1) | 7/476 (1.5) | 15/570 (2.6) |  |
| OR(95%CI) | 0.37 (0.08-1.68) | | 0.53 (0.11-2.65) | | 0.56 (0.23-1.38) | | 0.82 |
| **eGFR (mL/min/1.73 m²)** | | | | | | | |
| No.(%) | 14/114 (12.3) | 34/212 (16.0) | 32/177 (18.1) | 43/285 (15.1) | 54/476 (11.3) | 103/570 (18.1) |  |
| OR(95%CI) | 0.73 (0.38-1.42) | | 1.25 (0.77-2.03) | | 0.58 (0.41-0.81) | | 0.93 |

*Data presented as the number of patients/total number (%). Odd ratio (OR) are presented for CR versus ICR from a logistic regression model with the outcome of interest as the dependent variable and randomized treatment. Abbreviations: CR, complete revascularization; ICR, incomplete revascularization; eGFR, estimated glomerular filtration rate; LVEF, left ventricular ejection fraction.*
